# Temperature-Induced Plasmon Excitations for the α–$T_{3}$ Lattice in Perpendicular Magnetic Field

**DOI:** 10.3390/nano11071720

**Published:** 2021-06-29

**Authors:** Antonios Balassis, Godfrey Gumbs, Oleksiy Roslyak

**Affiliations:** 1 Department of Physics & Engineering Physics, Fordham University, 441 East Fordham Road, Bronx, NY 10458, USA; oroslyak@fordham.edu; 2 Department of Physics and Astronomy, Hunter College of the City University of New York, 695 Park Avenue, New York, NY 10065, USA; ggumbs@hunter.cuny.edu; 3 Donostia International Physics Center (DIPC), Paseo de Manuel Lardizabal 4, E-20018 San Sebastián, Spain

**Keywords:** magnetoplasmons, spin-1 fermions, dielectric function

## Abstract

We have investigated the α–$T_{3}$ model in the presence of a mass term which opens a gap in the energy dispersive spectrum, as well as under a uniform perpendicular quantizing magnetic field. The gap opening mass term plays the role of Zeeman splitting at low magnetic fields for this pseudospin-1 system, and, as a consequence, we are able to compare physical properties of the the α–$T_{3}$ model at low and high magnetic fields. Specifically, we explore the magnetoplasmon dispersion relation in these two extreme limits. Central to the calculation of these collective modes is the dielectric function which is determined by the polarizability of the system. This latter function is generated by transition energies between subband states, as well as the overlap of their wave functions.

## 1. Introduction

The α–$T_{3}$ model [1,2,3] is the most recent class of low-dimensional materials which are encouraging from a technological point of view [4]. In its pristine state, α–$T_{3}$ is gapless, and its band structure consists of a flat band along with a pair of relativistic low-energy linear subbands with zero effective mass Dirac fermions, like graphene [5]. However, the added presence of a flat band at the Dirac point causes α–$T_{3}$ to yield critical differences in its electronic and optical properties from those of graphene. This dispersionless energy band and the Dirac cones are affected by low magnetic field resulting in massive spin-1 quasiparticles near the K point. Here, we examine the way in which this mass term distorts the flat band and how the location of the Fermi energy in the presence of this distortion affects the plasmon excitation spectrum.

The difference between α–$T_{3}$ and the graphene honeycomb lattice arises from an added fermionic hub atom C at the center of each hexagon. Let the hopping integral be $t_{1}$ between the hub atom and either an A or B atom on the rim and $t_{2}$ between nearest neighbors on the rim of the hexagon. The ratio of these two nearest neighbor hopping terms is denoted as $t_{2}/t_{1}=\alpha$ where the parameter α satisfies 0≤α≤1. The largest value when α is 1 is for the dice lattice, whereas its value of 0 corresponds to graphene for decoupled hub from rim atoms. Some models with larger interactions having α>1 between the hub and rim atoms have recently been proposed and subsequently examined. The three-component Dirac-Weyl Hamiltonian with pseudospin 1 of an α–$T_{3}$ material is significantly different compared to that encountered for graphene and buckled structures, such as phosphorene, since we are dealing now with three-by-three matrices and inequivalent states for electrons and holes. Specifically, in the presence of magnetic field, we obtain the valley-dependent electron wave functions and even the energies, which enable valley filtering and other technical valleytronic application.

At first treated as a purely theoretical interpolation structure [6], the α–$T_{3}$ pseudospin-1 system of massless Dirac-Weyl fermions proposed by Bercioux et al. [6] has become a reality in a wide variety of naturally existing and artificially fabricated materials [7]. The novel electronic properties of the α–$T_{3}$ model have found incontrovertible experimental verification. This includes doped Hg${}_{1-x}$Cd${}_{x}$Te quantum wells [8], the tri-layer arrangement of SrTiO${}_{3}$/SrIrO${}_{3}$/SrTiO${}_{3}$ [9], Kagome [10,11,12] optical lattices and waveguides [13], and Lieb [14,15,16,17], as well as Josephson, arrays [18]. Ref. [19] offers a detailed review of dice-like systems having a flat band.

It has already been clearly demonstrated that a phase transition in α–$T_{3}$ occurs as the hopping parameter α approaches zero [20,21]. This change was shown to be driven by high magnetic field in the integer quantum Hall regime. In Ref. [20], it was demonstrated that, as the hopping parameter is continuously reduced to zero, there is a fundamental critical change in the behavior of the polarization function, which, in turn, leads to a softening of a magnetoplasmon mode. Furthermore, this critical behavior takes place in the K but not the K${}^{\prime }$ valley due to the remarkably different behaviors near these two symmetry points. Lastly, it was discovered that, in this high magnetic field limit, transitions from the flat band dominate in the K valley as the parameter α→0. We examine these behaviors in the low field regime, as well as at high magnetic fields, at finite temperature, in the absence of any doping.

We note that the combined effect of doping and temperature on low-energy plasmons in monolayer graphene has been investigated [22,23]. These works have revealed the anisotropy of the Coulomb excitations. Specifically, comparison was made for the π-plasmon dispersion relations when monolayer graphene is doped with carriers in the conduction band, and the temperature is zero, with the case when the temperature is finite, and there is no doping.

The remainder of this paper is organized as follows. In Section 2, we present the Hamiltonian for the α–$T_{3}$ model with a gap term. The eigenstates for this pseudospin-1 Hamiltonian are introduced for completeness. In Section 3, we present the polarization function, as well as the plasmon mode dispersion relation, in the presence of dispersive band gaps. Section 4 is devoted to the case when an applied perpendicular magnetic field is so strong that there is Landau quantization with only a few of the lowest energy subbands occupied for which we have calculated the temperature induced magnetoplasmon modes in addition to the polarization function. In Section 5, closed form analytic expressions are presented for the long wavelength magnetoplasmons in high magnetic field by employing the Kubo formula for the optical conductivity. We conclude with a summary of the key results in our paper for gapped α–$T_{3}$ in Section 6.

## 2. The Energy Spectrum at Low Magnetic Field

In the presence of a weak magnetic field, the low-energy Hamiltonian at the K and K${}^{\prime }$ point is [24,25,26]

(1) $${\hat{H}}_{\lambda }=\left(\begin{array}{ccc} \Delta & f(k)\cos\phi & 0\\ f^{*}\left(k\right)\cos\phi & 0 & f(k)\sin\phi \\ 0 & f^{*}\left(k\right)\sin\phi & -\Delta \end{array}\right).$$

In this equation, the origin of the k-space is specified to be around the K point, $k=(k_{x},k_{y})$ and $\tan\theta_{k}=k_{y}/k_{x}$, $\phi =\tan^{-1}\alpha$ in terms of the ratio of the hoping integrals defined above, $f\left(k\right)=\hbar v_{F}\left(\lambda k_{x}-ik_{y}\right)=\lambda \hbar v_{F}k\;e^{-i\lambda \theta_{k}}$, with λ=±1 being the valley index at the K and K${}^{\prime }$ points. The calculation is readily carried out analytically to obtain the eigenstates. These are the roots of the cubic equation

(2) $$\epsilon \left(\epsilon -\Delta \right)\left(\epsilon +\Delta \right)-{\left|f(k)\right|}^{2}\left[\epsilon +\Delta \cos(2\phi )\right]=0.$$

Write Equation (2) in the form $\epsilon^{3}-A\epsilon -B=0$, where $A=\Delta^{2}+{\left|f(k)\right|}^{2}$ and $B={\left|f(k)\right|}^{2}\Delta \cos\left(2\phi \right)$. To solve this cubic equation, let us compare it term-by-term with the trigonometric identity $\cos\left(3x\right)=4\cos^{3}x-3\cos x$. This leads to $\cos x=\pm \sqrt{3/A}\left(\epsilon /2\right)$ and $\cos\left(3x\right)=\left(3B/A\epsilon \right)\cos x$. Combining these results, we obtain the solution

(3) $$\begin{array}{ccc} \epsilon_{k} & = & {\left(\frac{4A}{3}\right)}^{1/2}\;\cos\theta \\ & = & {\left(\frac{4A}{3}\right)}^{1/2}\;\cos\left\{\frac{1}{3}\cos^{-1}\left[{\left(\frac{3}{A}\right)}^{3/2}\left(\frac{B}{2}\right)\right]+\frac{(s-1)\pi }{3}\right\},\;\;\;\;s=-1,0,1. \end{array}$$

This is a special case of the more general trinomial equation $x^{N}-x+t=0$, where N=2,3,…, whose solutions can be expressed as a finite sum of generalized hypergeometric functions [27,28], which can be seen to agree with Ref. [26]. We see from Equation (3) that we have three subbands, one for each distinct value of *s*.

Approximately, we have near ϵ=Δ

(4) $$\epsilon_{k}\approx \Delta +\frac{{\left|f(k)\right|}^{2}}{\Delta }\left(\frac{1}{1+\alpha^{2}}\right),$$

but, near ϵ=−Δ, we find that

(5) $$\epsilon_{k}\approx -\Delta -\frac{{\left|f(k)\right|}^{2}}{\Delta }\left(\frac{\alpha^{2}}{1+\alpha^{2}}\right),\;$$

clearly demonstrating that the magnetic field breaks the symmetry between the two subbands of electrons and holes. Additionally, close to ϵ=0, we no longer have a flat band but obtain

(6) $$\epsilon_{k}\approx -\frac{{\left|f(k)\right|}^{2}}{\Delta }\;\left(\frac{1-\alpha^{2}}{1+\alpha^{2}}\right).$$

These results show that there is no longer a flat band when we include the mass term Δ, that the conduction and valence bands are parabolic in the long wavelength region, and that the effective masses of the electron and hole near k=0 very much depend on the hopping parameter α.

The eigenfunctions for these subbands for the Hamiltonian (1) are given by the following:

(7) $$\psi_{k}\left(r\right)=\left(\begin{array}{c} \psi_{A}\left(k\right)\\ \psi_{B}\left(k\right)\\ \psi_{C}\left(k\right) \end{array}\right)\frac{e^{ik\cdot r}}{\sqrt{A}},$$

where *A* is a normalization area$,\;{\left|\psi_{A}\right|}^{2}+{\left|\psi_{B}\right|}^{2}+{\left|\psi_{C}\right|}^{2}=1$, and

(8) $$\begin{array}{ccc} \psi_{A}\left(k\right) & = & \left\{\frac{f(k)\cos\phi }{\epsilon_{k}-\Delta }\right\}\psi_{B}\left(k\right)\\ \psi_{C}\left(k\right) & = & \left\{\frac{f^{*}\left(k\right)\sin\phi }{\epsilon_{k}+\Delta }\right\}\psi_{B}\left(k\right). \end{array}$$

Therefore,

(9) $$\begin{array}{ccc} \psi_{B}\left(k\right) & = & |\epsilon_{k}-\Delta ||\epsilon_{k}+\Delta |\\ & \times & {\left\{{\left(\epsilon_{k}-\Delta \right)}^{2}{\left(\epsilon_{k}+\Delta \right)}^{2}+{\left(\epsilon_{k}+\Delta \right)}^{2}{\left|f(k)\right|}^{2}\cos^{2}\phi +{\left(\epsilon_{k}-\Delta \right)}^{2}{\left|f(k)\right|}^{2}\sin^{2}\phi \right\}}^{-1/2}. \end{array}$$

Consequently, these results show that although the energy bands are valley degenerate, and their eigenfunctions are not in the presence of the external field introduced by the parameter Δ. This feature plays a role in determining the dynamic polarizability.

**Figure 1:**
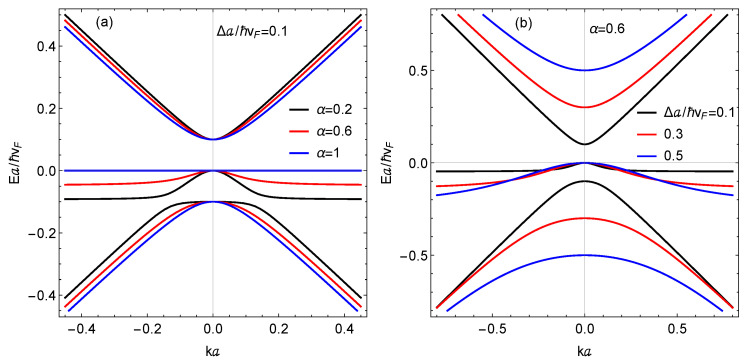
Energy dispersion (in units of $\hbar v_{F}/a$, where *a* is the lattice parameter) as a function of wave number (in units of 1/a) ) of the subbands determined by solving Equation (3) with the mass term having (**a**) $\Delta a/\hbar v_{F}=0.1$ and the hopping parameter α is 0.2, 0.6 and 1.0. In (**b**), we chose α=0.6 and calculated the subband spectra for various values of Δ in units of $\hbar v_{F}/a$.

In Figure 1, we present results for the three subbands in the presence of the mass term for various values of Δ. We used a=1.42 Å and $v_{F}=10^{6}$ m/s. We also carried out calculations for a range of the hopping parameter α. These results confirm Equations (4) and (5) that a gap 2Δ opens up between the electron and hole subbands at k=0, and their curvatures in the long wavelength limit are unequal and depend on α. The original flat subband now has negative curvature near k=0 in conformity with Equation (6). An important consequence of the energy gap between the electron, hole, and middle subbands is now the possible occurrence of dipolar excitons with the electrons and holes confined to parallel layers which are separated by a dielectric medium. Moreover, the gapped spin-1 α–$T_{3}$ may yield the co-existence of two types of dipolar excitons with electrons in the conduction band and holes in the valence or middle subband. The two-component superfluidity of these dipolar excitons may also be an interesting subject to investigate as it was done for both graphene [29] and phosphorene [30].

## 3. Low-Magnetic Field Case

### 3.1. Polarization Function in Low Magnetic Field

We now turn to another step in our investigation, which is the calculation of the the frequency ω and wave vector *q* dependent longitudinal polarization function. This is given by [8,31]

(10) $$\Pi \left(q,\omega \right)=g_{s}\sum_{\lambda =\pm }\sum_{s,s^{\prime }}\int \frac{d^{2}k}{{\left(2\pi \right)}^{2}}\frac{f\left(\epsilon_{s^{\prime },k+q}\right)-f\left(\epsilon_{s,k}\right)}{\hbar \omega +\epsilon_{s^{\prime },k+q}-\epsilon_{s,k}+i\delta }F_{s,k,s^{\prime },k+q}^{\lambda \lambda^{\prime }}\left(q\right),$$

where $g_{s}=2$ is the spin degeneracy, $f(\epsilon_{s,k})$ is the Fermi-Dirac distribution function, and $F_{s,k,s^{\prime },k+q}^{\lambda }\left(q\right)$ is an overlap function

(11) $$F_{s,k,s^{\prime },k+q}^{\lambda \lambda^{\prime }}\left(q\right)\equiv {\left|\langle \psi_{s,k}^{\lambda }|\psi_{s^{\prime },k+q}^{\lambda^{\prime }}\rangle \right|}^{2}.$$

As a matter of fact, the evaluation of the overlap factor in Equation (11) can be carried out analytically for arbitrary ϕ when Δ=0 and use is made of the wave functions in the absence of the mass term, which are

(12) $$\psi_{k}^{\left(0\right)}\left(r\right)=\left(\begin{array}{c} -\exp\left(-i\lambda \theta_{k}\right)\sin\phi \\ 0\\ \exp\left(i\lambda \theta_{k}\right)\cos\phi \end{array}\right)\frac{e^{ik\cdot r}}{\sqrt{A}},$$

with eigenvalue $\epsilon_{s}\left(k\right)=0$ for the flat band, and

(13) $$\psi_{k}^{\left(s\right)}\left(r\right)=\frac{1}{\sqrt{2}}\left(\begin{array}{c} \left(\lambda /s\right)\exp\left(-i\lambda \theta_{k}\right)\cos\left(\phi \right)\\ 1\\ \left(\lambda /s\right)\exp\left(i\lambda \theta_{k}\right)\sin\left(\phi \right) \end{array}\right)\frac{e^{ik\cdot r}}{\sqrt{A}},$$

with eigenvalue $\epsilon_{s}\left(k\right)=s\hbar v_{F}\left|k\right|$, where s=+1 for the conduction band, and s=−1 for the valence band. After some algebra, we obtain for the case when Δ=0

(14) $$\langle 0\lambda ;k|s^{\prime }\lambda^{\prime };k^{\prime }\rangle =-i\frac{1}{\sqrt{2}}\left(\frac{\lambda^{\prime }}{s^{\prime }}\right)\sin\left(2\phi \right)\sin\left(\lambda \theta_{1}-\lambda^{\prime }\theta_{2}\right)$$

and

(15) $$\langle s\lambda ;k|s^{\prime }\lambda^{\prime };k^{\prime }\rangle =\frac{1}{2}\left\{1+\left(\frac{\lambda }{s}\right)\left(\frac{\lambda^{\prime }}{s^{\prime }}\right)\left[e^{i(\lambda \theta_{1}-\lambda^{\prime }\theta_{2})}\cos^{2}\left(\phi \right)+e^{-i(\lambda \theta_{1}-\lambda^{\prime }\theta_{2})}\sin^{2}\left(\phi \right)\right]\right\},$$

where the wave vectors are $k=(k,\theta_{1})$ and $k^{\prime }=\left(k^{\prime },\theta_{2}\right)$. For overlap of the eigenstates within the same valley, i.e., $\lambda =\lambda^{\prime }$, the results in Equations (14) and (15) can be expressed in terms of the angle $\Theta_{k}=\theta_{1}-\theta_{2}$ between k and $k^{\prime }$, which was exploited in Ref. [8] in the calculation of the polarizability of the translationally invariant model for the α–$T_{3}$ model.

**Figure 2:**
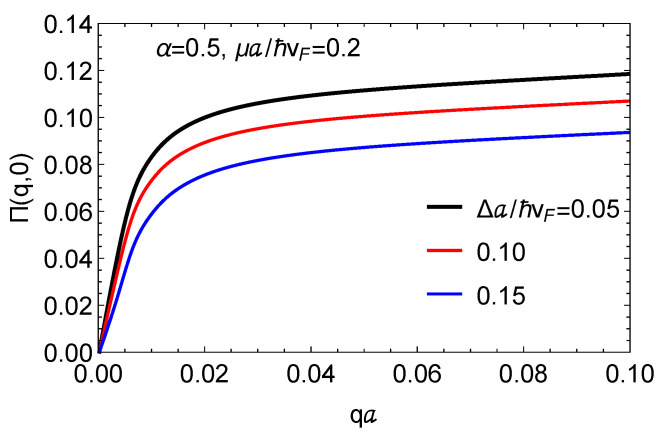
Plots for the static polarization function at *T* = 0 K as a function of wave number in units of the inverse lattice constant for chosen $\mu =0.2\hbar v_{F}/a$ and hopping parameter α=0.5. The results are compared for various mass terms Δ.

In Figure 2, we present the static polarization function at zero temperature for various mass term parameters Δ. The hopping ratio α is fixed at 0.5, and the chemical potential is set at $\mu =0.2\;\hbar v_{F}/a$. These results show that, as the value of the mass term is increased, the static polarization is decreased. This dependence allows for the tunability of static charge screening by gapped α–$T_{3}$ materials, as well as image states, which may be probed by photoemission experiments.

### 3.2. Plasmon Dispersion in Low Magnetic Fields

Making use of the polarization function, we have calculated the plasmon mode dispersion relation numerically for magnetoplasmons for the α–$T_{3}$ model in the presence of a uniform perpendicular magnetic field for various values of the coupling parameter α. These correspond to the resonance of the polarizability for interacting electrons which, in the random-phase approximation (RPA), is given by

(16) $$\Pi^{RPA}\left(q,\omega \right)=\frac{\Pi (q,\omega )}{1-v(q)\Pi (q,\omega )}\equiv \frac{\Pi (q,\omega )}{\epsilon (q,\omega )},$$

where v(q) is the Coulomb potential.

**Figure 3:**
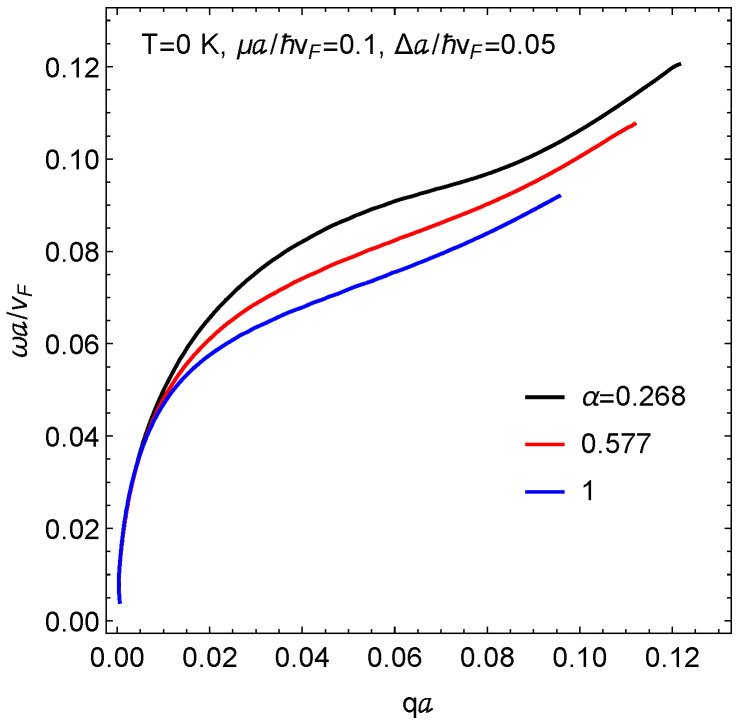
Plasmon dispersion as a function of the wave number for three values of the hopping parameter α and fixed mass parameter Δ. The Fermi energy is within the conduction band.

**Figure 4:**
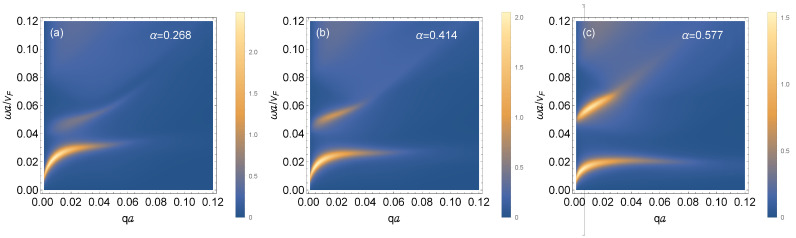
Density plots of the magnetoplasmons dispersion for an α–$T_{3}$ lattice in low magnetic field for chosen temperature *T* = 0 K, mass term $\Delta a/\hbar v_{F}=0.05$ and various values of the hopping parameter α, (**a**) α=0.268, (**b**) α=0.414 and (**c**) α=0.577. The Fermi energy $\mu a/\hbar v_{F}=-0.0125$ is within the middle band.

Figure 3 and Figure 4 present the plasmon dispersion at zero temperature using the value $e^{2}/\left(4\pi \epsilon_{0}\hbar v_{F}\right)\approx 2.2$. Figure 3 shows plasmon branches when the chemical potential is located in the conduction band for various values of the hopping parameter α. In each case, the plasmon branch is terminated due to Landau damping by the single-particle-excitations. The branch corresponding to the dice lattice, i.e., α=1, is Landau damped at a smaller wave vector than the one closer to the graphene limit (α→0). However, all three branches have the same group velocity in the long wavelength limit, showing that the presence of a gap has virtually no effect on the Coulomb excitations in this regime.

Figure 4 shows the plasmon dispersion when the chemical potential is placed in the “deformed” flat band, which has a local maximum when α<1 at k=0, as seen in Figure 1, for various α. But, when α=1, the flat band is dispersionless. In all three panels of Figure 4, there are two plasmon branches. This is due to the allowed transitions between (i) the valence and conduction bands and (ii) the modified flat band and the conduction band. Interestingly, the intensity of the lower branch (valence to conduction band) remains the same in Figure 4a–c as α is varied. However, the intensity of the upper branch (middle to conduction) is increased as α is increased. As Equation (6) shows, the middle band is dispersionless when α=1. This is in agreement with Figure 1a. Therefore, when α=1, the density-of-states in the middle band from which transitions take place is infinite, thereby giving rise to a brighter plasmon branch.

**Figure 5:**
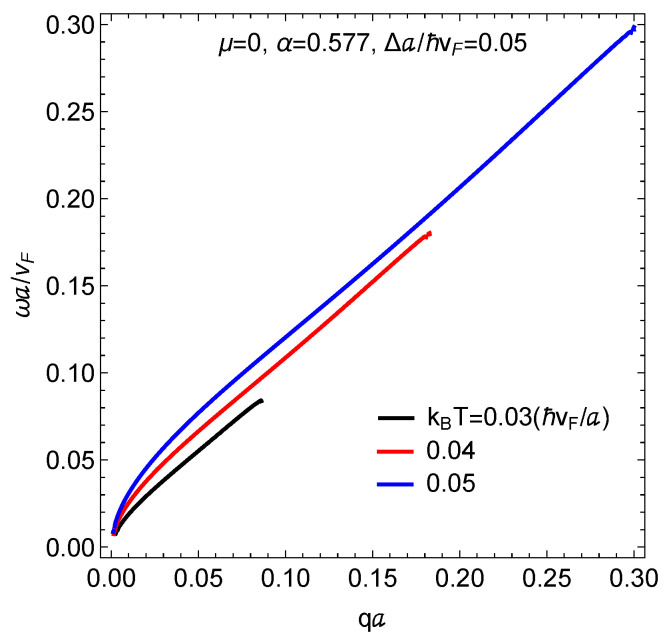
Frequency versus wave number of the temperature-induced magnetoplasmons in an α–$T_{3}$ model in low magnetic field. Three different temperatures are chosen. The hopping and mass term parameters are listed.

In Figure 5, we present the results we have obtained for the temperature-induced magnetoplasmons for the gapped α–$T_{3}$ model with a mass term corresponding to $\Delta a/\hbar v_{F}=0.05$. Clearly, the plasmon mode frequency is increased as the temperature is increased since the Fermi tail becomes more far-reaching in the higher subband states. Additionally, the smearing of the Fermi surface at finite temperature causes the group velocity in the long wavelength limit to be reduced compared to its zero-temperature counterpart in Figure 3 where there is doping. These results also show that each magnetoplasmon branch is Landau damped at varying values of wave vector which depends on temperature, as well as the hopping and gap parameters.

## 4. High Magnetic Field Case

### 4.1. Polarization Function in High Magnetic Field

**Figure 6:**
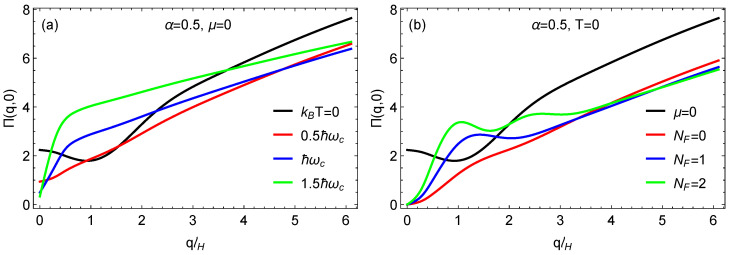
Plots for the static polarization function for (**a**) chosen μ=0, as well as selected temperatures and (**b**) zero temperature with filling factors $N_{F}$, which is the index of the highest occupied Landau level at *T* = 0 K defining the chemical potentials. The magnetic length is denoted by $l_{H}$ and the cyclotron frequency by $\omega_{c}$.

**Figure 7:**
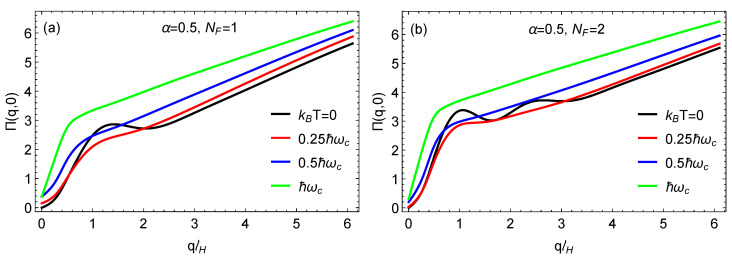
Plots for the static polarization for various temperatures and two values for (**a**) $N_{F}=1$ and (**b**) $N_{F}=2$, which refers to the index of the highest occupied Landau level at *T* = 0 K. The magnetic length is denoted by $l_{H}$ and the cyclotron frequency by $\omega_{c}$.

In the high magnetic field limit, Landau levels are formed and dominate over the mass term. In this regime, one must use Landau level wave functions and energies in the polarization function. Here, we consider temperature-induced Coulomb excitations in the specific case when there is integer filling at *T* = 0 K. The frequency ω and wave vector *q* dependent longitudinal polarization function is given in this case by [20]

(17) $$\Pi \left(q,\omega \right)=g_{s}\sum_{\lambda }\sum_{s,s^{\prime }}\sum_{n,n^{\prime }}\frac{f\left(\epsilon_{s^{\prime },n^{\prime }}^{\lambda }\right)-f\left(\epsilon_{s,n}^{\lambda }\right)}{\hbar \omega +\epsilon_{s^{\prime },n^{\prime }}^{\lambda }-\epsilon_{s,n}^{\lambda }+i\delta }F_{sn,s^{\prime }n^{\prime }}^{\lambda }\left(q\right),$$

where the energy states are given by $\epsilon_{s,n}^{\lambda }=sv_{F}\sqrt{2\hbar eB}\sqrt{n+\chi_{\lambda }}$, $f(\epsilon_{s,n})$ is the Fermi-Dirac distribution function, and the form factor is given by $F_{sn,s^{\prime }n^{\prime }}^{\lambda }\left(q\right)\equiv |<\psi_{sn}^{\lambda }|e^{iq\cdot r}{\left|\psi_{s^{\prime }n^{\prime }}^{\lambda }>\right|}^{2}$. The auxilary parameter $\chi_{\lambda }=\left[1-\lambda \cos\left(2\phi \right)\right]/2$ has also been used. At zero temperature, we have $f\left(\epsilon_{s,n}\right)=\theta \left(\mu -\epsilon_{s,n}\right)$ in terms of the Heaviside step function. In Figure 6 and Figure 7, we present our results for the static polarization function versus the wave vector *q*. We use the magnetic length $l_{H}=\sqrt{\hbar /(eB)}$ and the cyclotron frequency $\omega_{c}=\sqrt{2}v_{F}/l_{H}$. In Figure 6a, we chose α=0.5, and, with the Fermi level in the flat band, we explore the behavior at various temperatures. At *T* = 0 K, there is no peak in the polarization, but, as the temperature is raised, a well defined peak emerges, which is clearly visible at $T=1.5\;T_{B}$, where $k_{B}T_{B}=\hbar \omega_{c}$. To interpret the significance of this shape, we turn to Figure 6b, where *T* = 0 K, and the number of occupied Landau levels is varied. When the Fermi level is within the flat band at zero temperature (μ=0), the polarization function has no peak, but this changes as the Fermi level is raised, and the number of peaks corresponds to the occupation number index $N_{F}$. Therefore, we conclude that finite temperature in Figure 6a simulates the Landau level occupation which we impose in Figure 6b. In Figure 7a,b, we again chose α=0.5 for comparison with the results in Figure 6. These results show how the *T* = 0 K peaks for $N_{F}=1$ and $N_{F}=2$ are smeared as the temperature is increased, eventually disappearing at sufficiently high temperature.

### 4.2. Magnetoplasmons in High Magnetic Field

**Figure 8:**
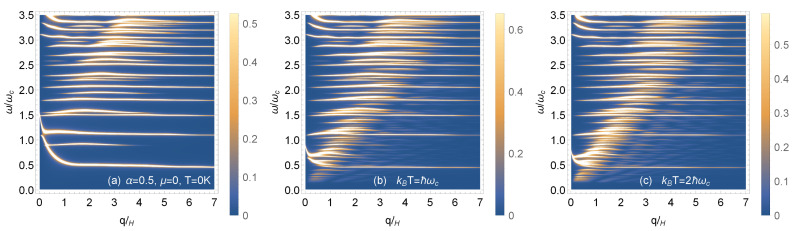
Density plots of the magnetoplasmon dispersion relation for various temperatures (**a**–**c**). The hopping parameter is α=0.5 and the chemical potential, μ=0, is set at the top of the flat band.

**Figure 9:**
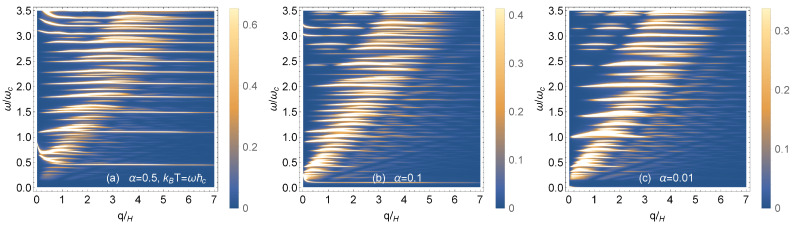
Density plots of the magnetoplasmon dispersion relation for various hopping parameters (**a**) α=0.5, (**b**) α=0.1, and (**c**) α=0.01. The temperature is chosen as $k_{B}T=\hbar \omega_{c}$.

In Figure 8 and Figure 9, we compare the magnetoplasmon dispersion relations in high magnetic field. In all cases, the Fermi level is chosen so that there is integer filling of the Landau levels via an applied gate voltage. In Figure 8a–c, there is no doping, i.e., μ=0 at *T* = 0 K. We chose zero temperature and two finite temperatures. The depolarization shifts in the long wavelength limit are largest for the low frequency modes, but these shifts are reduced as the temperature is increased. The intensity of the high frequency modes is decreased at larger values of the wave number. Figure 9a–c show how the magnetoplasmon modes are affected as the hopping parameter α is reduced at chosen finite temperature. The lowest magnetoplasmon mode is softened, i.e., gets lower and lower in frequency and eventually becomes undetectable as α→0.

## 5. Magneto-Plasmons in α–$T_{3}$ Lattice via the Transfer Matrix Approach

Long wavelength plasmon dispersion can be calculated via the transfer matrix approach [32,33]. For a single conducting interface in vacuum, the generic dispersion relation for transverse electric (TE) and (TM) magnetoplasmons is given by

(18) $$\begin{array}{c} q_{TM,TE}=\frac{i\omega \left[4-\sigma_{xy}\sigma_{yx}+\sigma_{xx}\sigma_{yy}\pm \sqrt{{\left(4-\sigma_{xy}\sigma_{yx}+\sigma_{xx}\sigma_{yy}\right)}^{2}-16\sigma_{xx}\sigma_{yy}}\right]}{4\sigma_{xx}}, \end{array}$$

where the TM (TE) subscripts correspond to the + (−) signs, respectively. The TE waves for our particular system yield $q_{TE}<0$ and are, therefore, non-physical in the non-retarded limit.

The optical conductivity in Equation (18) is given by the Kubo formula

(19) $$\begin{array}{c} \sigma_{\alpha \beta }=\frac{i\sigma_{0}}{2\pi }\sum_{n,n^{\prime }}\frac{\theta \left(\mu_{F}-\epsilon_{n}\right)-\theta \left(\mu_{F}-\epsilon_{n^{\prime }}\right)}{\epsilon_{n^{\prime }}-\epsilon_{n}}\\ \times \frac{\langle \Psi_{n}{\hat{j}}_{\alpha }\Psi_{n^{\prime }}\Psi_{n^{\prime }}{\hat{j}}_{\beta }\Psi_{n}\rangle }{\omega -\left(\epsilon_{n^{\prime }}-\epsilon_{n}\right)+i\gamma }, \end{array}$$

where $\sigma_{0}=e^{2}g/\left(2\hbar \right)$ is the universal conductivity, and all energies are normalized by the magnetic energy $\gamma_{B}=v_{F}\sqrt{2\hbar eB}$ and are given by

(20) $$\epsilon_{n}=s\sqrt{n-\frac{1}{2}-\frac{\lambda }{2}\cos2\phi }.$$

In this notation, the composite index n={λ,s,n} labels λ=±1 as the valley index, s=0,±1 is the subband index, and integer *n* is the Landau level index; ${\hat{j}}_{\alpha }=v_{F}^{-1}(\partial \hat{H}/\partial k_{\alpha )}$ is the normalized current operator given via the derivatives of the α–$T_{3}$ Hamiltonian along the α=x,y directions. Based on the symmetry of the problem, we have $\sigma_{yy}=\sigma_{xx}$ and $\sigma_{yx}=-\sigma_{xy}$.

The current products for the valence to conduction band transitions $s=-s^{\prime }=\pm 1$ may be written in terms of auxiliary functions:

(21) $$\begin{array}{c} F_{1,s,n}^{1,s^{\prime },n^{\prime }}=\frac{s}{4}\cos\phi \sqrt{\frac{n\cos^{2}\phi }{n+1-\cos^{2}\phi }}\\ +\frac{s^{\prime }}{4}\sin\phi \sqrt{\frac{n^{\prime }\sin^{2}\phi }{n^{\prime }-\cos^{2}\phi }}\\ F_{-1,s,n}^{-1,s^{\prime },n^{\prime }}=\frac{s}{4}\sin\phi \sqrt{\frac{n\sin^{2}\phi }{n+1-\sin^{2}\phi }}\\ +\frac{s^{\prime }}{4}\cos\phi \sqrt{\frac{n^{\prime }\cos^{2}\phi }{n^{\prime }-\sin^{2}\phi }}. \end{array}$$

The resulting current overlaps are

(22) $$\begin{array}{c} J_{\lambda ,xx}^{s,n,s^{\prime },n^{\prime }}=\langle \Psi_{\lambda ,s,n}|{\hat{j}}_{x}|\Psi_{\lambda ,s^{\prime },n^{\prime }}\rangle \langle \Psi_{\lambda ,s^{\prime },n^{\prime }}|{\hat{j}}_{x}|\Psi_{\lambda ,s,n}\rangle \\ =F_{\lambda ,s^{\prime },n}^{\lambda ,s,n}\delta_{n^{\prime },n+1}+F_{\lambda ,s^{\prime },n-1}^{\lambda ,s,n-1}\delta_{n^{\prime },n-1} \end{array}$$

(23) $$\begin{array}{c} J_{\lambda ,xy}^{s,n,s^{\prime },n^{\prime }}=\langle \Psi_{\lambda ,s,n}|{\hat{j}}_{x}|\Psi_{\lambda ,s^{\prime },n^{\prime }}\rangle \langle \Psi_{\lambda ,s^{\prime },n^{\prime }}|{\hat{j}}_{y}|\Psi_{\lambda ,s,n}\rangle \\ =-iF_{\lambda ,s,n}^{\lambda ,s^{\prime },n}\delta_{n^{\prime },n+1}+iF_{\lambda ,s^{\prime },n-1}^{\lambda ,s,n-1}\delta_{n^{\prime },n-1}. \end{array}$$

The rest of the current products are provided in Appendix A.

**Figure 10:**
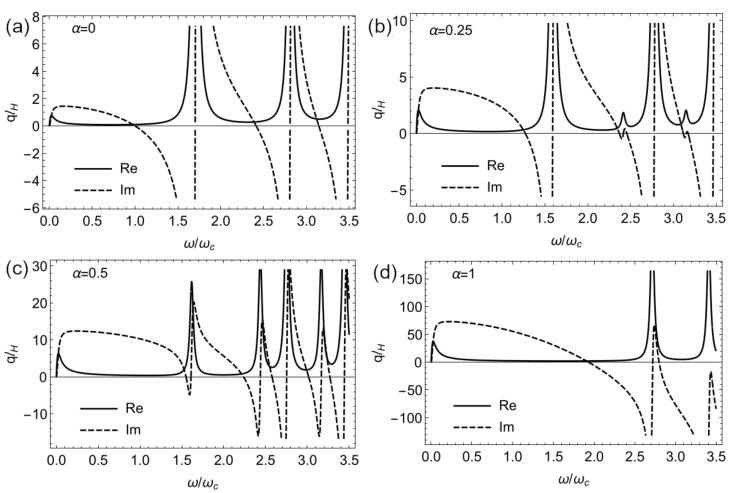
Real and imaginary parts of the wavevector for TM plasmons in the long wavelength approximation for μ=0, (**a**) α=0, (**b**) α=0.25 and (**c**) α=0.5 and (**d**) α=1. Setting $\sigma_{xy}=0$ has no effect on the dispersion due to the inherited isotropy of the structure.

In order to keep things simple, let us consider the case when there is no doping with μ=0 and compare the plasmon modes with Figure 8a. In this case, the magnetoplasmons are formed via collective excitations due to inter-band transitions as revealed in Figure 10. Since we study the case where $q_{TM}\to 0$, any further discussion must be restricted to a few plasmon branches satisfying this condition.

For α=0, we note that the long-range self-sustaining plasmon modes correspond to vanishing imaginary part of $ql_{H}$ and given by the selection rules in Equation (5). The lowest energy plasmon excitations correspond to the transition $\Omega_{c}\left(0\right)=\epsilon_{n^{\prime }}-\epsilon_{n}=\epsilon_{1,1,1}-\epsilon_{1,-1,2}=\omega_{c}$, as shown in the corresponding panel of Figure 10. For a small increase in the parameter α, the effective cyclotron frequency is blue shifted $\Omega_{c}\left(\alpha \right)=\omega_{c}\left(1+\tan^{-1}\alpha \right)$ and is effectively doubled at α=1. The next plasmon branch satisfying the long wavelength approximation corresponds to the inter-band transitions in the K${}^{{}^{\prime }}$ valley with $\Omega_{c}\left(\alpha \right)=\epsilon_{-1,1,1}-\epsilon_{-1,-1,2}=\omega_{c}\left[2.41-0.85{\left(\tan^{-1}\alpha \right)}^{2}\right]$. All the plasmon branches described in the long wavelength approximation are also present in Figure 8a.

## 6. Concluding Remarks and Summary

In this paper, we have theoretically investigated the role played by an ambient magnetic field on self-sustained plasma excitations for both a doped and undoped α–$T_{3}$ model. Two distinct regimes are studied. In the low-field limit, we simulate the role of magnetic field by adding a mass term to the Hamiltonian, which introduces an energy gap between the Dirac bands, but, at high magnetic fields, Landau level quantization is introduced. We employed the energy subband structure and wave functions to calculate the polarization function, which, in turn, was used to determine the longitudinal dielectric function. Crucial to the properties of the polarizability of α–$T_{3}$ are the allowed single-particle transitions, which are determined by the wave function overlap or form factor.

For undoped α–$T_{3}$ with a mass term, the position of the Fermi level could give rise to characteristically different plasmon mode spectra. Referring to Figure 1, there are two energy gaps: one between the conduction and middle band and one between the middle band and the valence band. In Figure 4b, we observe two plasmon branches, which arise by design when the Fermi energy is located within the middle band, allowing possible transitions from within the valence and middle band to the conduction band. Comparing Figure 4a,b, we conclude that the mode with the higher frequency has its intensity reduced as α is reduced, i.e., this mode becomes soft as the parameter α→0 where a phase transition occurs. The softening of low-frequency magnetoplasmon modes also occurs near this phase transition.

Our theoretical investigation into the Coulomb excitations of α–$T_{3}$ requires the input of a key quantity, i.e., the polarization function. Contributing to the polarizability are the excitations between states. However, they can only subscribe depending on the occupation and/or availability of states as described by the difference in Fermi distribution functions appearing in Equations (10) and (17). But, appearing alongside the Fermi functions is the form factor describing the overlap of the wave functions for the states involved in the elementary excitations.

Finally, the plasmon modes we describe in this work should be successfully probed experimentally with the use of inelastic electron scattering or electron energy-loss spectroscopy (EELS) [34,35] compared with photon-based spectroscopies. It turns out that the spatial resolution of these optical spectroscopies is limited by their respective photon wavelengths, as well as other factors [36,37]. EELS has been the standard method for investigating the collective excitations of electrons, i.e., plasmons. In particular, the plasmon dispersion on epitaxial graphene was investigated using high-resolution EELS [38]. Over the years, this method has been improved, capable of subangstrom spatial resolution, to provide information on interband transitions between the valence and conduction bands, as well as on core-level excitations. These latter excitations provide information regarding the unoccupied density-of-states, as well as regarding the lattice structure. We are predicting significant differences in the collective modes with graphene due to the presence of the flat band. For example, at small momentum transfer *q*, a high-frequency plasmon could be excited when a gap opens up between the valence and conduction bands to allow the Fermi level to lay within the distorted flat band, thereby allowing interband transitions between the middle and conduction bands. Using high-resolution EELS, this mode should be observed.

## Data Availability

The data can be available at request from the authors.

## Appendix A

The current-current products $J_{\lambda ,\alpha \beta }^{s,n,s^{\prime },n^{\prime }}=\langle \lambda ,s,n|\hat{j_{\alpha }}|\lambda ,s^{\prime },n^{\prime }\rangle \langle \lambda ,s^{\prime },n^{\prime }|\hat{j_{\beta }}|\lambda ,s,n\rangle$ for the K-valley (λ=1), when $s,s^{\prime }=\pm 1$ (valence to conduction band) are given by

(A1) $$\begin{array}{c} J_{\lambda =1,xx}^{s,n,s^{\prime },n^{\prime }}=J_{\lambda =1,yy}^{s,n,s^{\prime },n^{\prime }}=\frac{1}{4}{\left(s\cos^{2}\phi \sqrt{\frac{n}{n+1-\cos^{2}\phi }}+s^{\prime }\sin^{2}\phi \sqrt{\frac{n}{n-\cos^{2}\phi }}\right)}^{2}\delta_{n^{\prime },n+1}\\ +\frac{1}{4}{\left(s^{\prime }\cos^{2}\phi \sqrt{\frac{n-1}{n-\cos^{2}\phi }}+s\sin^{2}\phi \sqrt{\frac{n-1}{n-1-\cos^{2}\phi }}\right)}^{2}\delta_{n^{\prime },n-1}, \end{array}$$

whereas, when s=0 and $s^{\prime }=\pm 1$ (flat band to conduction/valence band), we have

(A2) $$J_{\lambda =1,xx}^{0,n,s^{\prime },n^{\prime }}=J_{\lambda =1,yy}^{0,n,s^{\prime },n^{\prime }}=\left[\frac{\left(n-1\right)\sin^{2}\phi \cos^{2}\phi }{2(n-\cos^{2}\phi )}\right]\delta_{n^{\prime },n+1}+\left[\frac{n\sin^{2}\phi \cos^{2}\phi }{2\left(n-\cos^{2}\phi \right)}\right]\delta_{n^{\prime },n-1}.$$

For the case when s=±1 and $s^{\prime }=0$ (conduction/valence band to flat band), we obtain

(A3) $$J_{\lambda =1,xx}^{s,n,0,n^{\prime }}=J_{\lambda =1,yy}^{s,n,0,n^{\prime }}=\left[\frac{\left(n^{\prime }-1\right)\sin^{2}\phi \cos^{2}\phi }{2\left(n^{\prime }-\cos^{2}\phi \right)}\right]\delta_{n^{\prime },n-1}+\left[\frac{n^{\prime }\sin^{2}\phi \cos^{2}\phi }{2(n^{\prime }-\cos^{2}\phi )}\right]\delta_{n^{\prime },n+1}.$$

We note that $J_{\lambda =1,xx}^{s,n,0,n^{\prime }}=J_{\lambda =1,xx}^{0,n^{\prime },s^{\prime },n}$, and the same relation holds for the yy currents.

For the off-diagonal current-current products, we have, in the case of valence-conduction band transitions,

(A4) $$\begin{array}{c} J_{\lambda ,xy}^{s,n,s^{\prime },n^{\prime }}=-\frac{i}{4}{\left(s\cos^{2}\phi \sqrt{\frac{n}{n+1-\cos^{2}\phi }}+s^{\prime }\sin^{2}\phi \sqrt{\frac{n}{n-\cos^{2}\phi }}\right)}^{2}\delta_{n^{\prime },n+1}\\ +\frac{i}{4}{\left(s^{\prime }\cos^{2}\phi \sqrt{\frac{n-1}{n-\cos^{2}\phi }}+s\sin^{2}\phi \sqrt{\frac{n-1}{n-1-\cos^{2}\phi }}\right)}^{2}\delta_{n^{\prime },n-1}, \end{array}$$

and $J_{\lambda ,yx}^{s,n,s^{\prime },n^{\prime }}=-J_{\lambda ,xy}^{s,n,s^{\prime },n^{\prime }}$. In the case of valence/conduction band (s=±1) to flat band ($s^{\prime }=0$) transitions, we obtain

(A5) $$J_{\lambda ,xy}^{s,n,s^{\prime }=0,n^{\prime }}=\frac{i\left(n+1\right)\sin^{2}\left(2\phi \right)}{8\left[\cos^{2}\phi -\left(n+1\right)\right]}\delta_{n^{\prime },n+1}-\frac{i\left(n-2\right)\sin^{2}\left(2\phi \right)}{8\left[\cos^{2}\phi -\left(n-1\right)\right]}\delta_{n^{\prime },n-1},$$

while, for flat band (s=0) to the conduction/valence band ($s^{\prime }=\pm 1$) transitions, we obtain

(A6) $$J_{\lambda ,xy}^{s=0,n,s^{\prime },n^{\prime }}=\frac{i\left(n^{\prime }+1\right)\sin^{2}\left(2\phi \right)}{8\left[\left(n^{\prime }+1\right)-\cos^{2}\phi \right]}\delta_{n^{\prime },n-1}-\frac{i\left(n^{\prime }-2\right)\sin^{2}\left(2\phi \right)}{8\left[\left(n^{\prime }-1\right)-\cos^{2}\phi \right]}\delta_{n^{\prime },n+1}.$$

We note that $J_{\lambda ,xy}^{s=0,n,s^{\prime },n^{\prime }}=-J_{\lambda ,xy}^{s,n,s^{\prime }=0,n^{\prime }}$.

For the K${}^{\prime }$-valley, the valence-conduction band transition matrix elements are given by

(A7) $$\begin{array}{c} J_{\lambda =-1,xx}^{s,n,s^{\prime },n^{\prime }}=J_{\lambda =-1,yy}^{s,n,s^{\prime },n^{\prime }}=\frac{1}{4}{\left(s\sin^{2}\phi \sqrt{\frac{n}{n+1-\sin^{2}\phi }}+s^{\prime }\cos^{2}\phi \sqrt{\frac{n}{n-\sin^{2}\phi }}\right)}^{2}\delta_{n^{\prime },n+1}\\ +\frac{1}{4}{\left(s^{\prime }\sin^{2}\phi \sqrt{\frac{n-1}{n-\sin^{2}\phi }}+s\cos^{2}\phi \sqrt{\frac{n-1}{n-1-\sin^{2}\phi }}\right)}^{2}\delta_{n^{\prime },n-1}, \end{array}$$

and we note that it can be obtained from the corresponding equation, Equation (A1), for the K-valley by the replacement sinϕ→cosϕ and cosϕ→sinϕ. In general, all the transition elements for the K${}^{\prime }$-valley can be obtained by this replacement into the corresponding elements for the K-valley.
